# Fumonisins in Conventional and Transgenic, Insect-Resistant Maize Intended for Fuel Ethanol Production: Implications for Fermentation Efficiency and DDGS Co-Product Quality

**DOI:** 10.3390/toxins6092804

**Published:** 2014-09-22

**Authors:** Erin L. Bowers, Gary P. Munkvold

**Affiliations:** 1 Department of Agricultural and Biosystems Engineering, National Swine Research Center, Iowa State University, Ames, IA 50011, USA; 2 Department of Plant Pathology and Microbiology, Seed Science Center, Iowa State University, Ames, IA 50011, USA; E-Mail: munkvold@iastate.edu

**Keywords:** fumonisin, distillers grains, DDGS, ethanol, fermentation, *Bacillus thuringiensis*, *Bt* maize, GM maize

## Abstract

Mycotoxins in maize grain intended for ethanol production are enriched in co-product dried distiller’s grains and solubles (DDGS) and may be detrimental to yeast in fermentation. This study was conducted to examine the magnitude of fumonisin enrichment in DDGS and to analyze the impacts of insect injury, Fusarium ear rot severity, and fumonisin contamination on final ethanol yield. Samples of naturally-contaminated grain (0 to 35 mg/kg fumonisins) from field trials conducted in 2008–2011 were fermented and DDGS collected and analyzed for fumonisin content. Ethanol yield (determined gravimetrically) was unaffected by fumonisins in the range occurring in this study, and was not correlated with insect injury or Fusarium ear rot severity. Ethanol production was unaffected in fumonisin B_1_-spiked grain with concentrations from 0 to 37 mg/kg. *Bacillus thuringiensis* (*Bt*) maize often has reduced fumonisins due to its protection from insect injury and subsequent fungal infection. DDGS derived from *Bt* and non-*Bt* maize averaged 2.04 mg/kg and 8.25 mg/kg fumonisins, respectively. Fumonisins were enriched by 3.0× for 50 out of 57 hybrid × insect infestation treatment combinations; those seven that differed were <3.0 (1.56 to 2.56×). This study supports the industry assumption of three-fold fumonisin enrichment in DDGS, with measurements traceable to individual samples. Under significant insect pest pressures, DDGS derived from *Bt* maize hybrids were consistently lower in fumonisins than DDGS derived from non-*Bt* hybrids.

## 1. Introduction

The maize ethanol industry continues to grow steadily in the United States, keeping in step with federal government directives for the inclusion of renewable fuels in the U.S. fuel supply. The Energy Policy Act of 2005 [1] and the Energy Independence and Security Act of 2007 [2] bolstered mandates for biofuel inclusion and directed that maize-based ethanol should constitute 57 billion liters (15 billion gallons) of the U.S. fuel supply annually by the year 2015. As of 2013, 211 maize ethanol plants are operating in 28 states with a total production capacity of 55.65 billion L (14.7 billion gallons) annually [3]. Ethanol plants are concentrated in the Central U.S., in close proximity to the country’s most intense maize production. In 2011, nearly 53 billion L (14 billion gallons) of maize-based ethanol were produced and utilized, and maize-derived ethanol constituted 10% of the total U.S. fuel consumption [4]. Additionally, gasoline/ethanol blends continue to comprise 95% of the total fuel consumed in the United States, the majority of which is a blend of 10% ethanol in gasoline.

Maize grain has long been considered a well-suited substrate for ethanol production as a result of the high starch content in maize kernels. Industrial-scale production of fuel ethanol is accomplished through one of two processes: dry grind or wet milling. Approximately 80% of ethanol plants currently operating use dry-grind processing methods. In dry grind processing, whole kernel maize is ground and mixed with water to form mash. The addition of heat and specific amylase enzymes enables the liquefaction of starch molecules into dextrins. The mixture is cooled, and a glucoamylase enzyme and yeast are added. Over a time frame of 48–72 h, the dextrins are saccharified and the resulting glucose fermented to produce ethanol and co-product, carbon dioxide. Ethanol is collected through distillation, and the remaining slurry contains solid material which is nutritionally valuable for livestock. Depending on processing, this feedstock is made available in the form of wet distiller’s grains, dried distiller’s grains or, most commonly, dried distiller’s grains and solubles (DDGS). Commercial dry grind ethanol processing yields approximately 0.42 L of ethanol and 7.7–8.2 kg of DDGS per 25 kg maize (2.8 gallons and 17–18 lbs. per bushel of maize at 15% moisture).

As a result of the predominance of dry grind over wet mill processing, co-production of DDGS has reached 30 million tons annually. DDGS are a valuable livestock feed component and their sale is vital for the profitability of the ethanol industry. DDGS quality can be affected by maize grain quality which can be compromised by insect feeding, fungal infection, and mycotoxin accumulation both in the field and in storage. Maize can suffer contamination by a number of mycotoxins including fumonisins, which are the most common mycotoxins found in areas of the U.S. with the highest ethanol production capacity. Fumonisins are produced primarily by *Fusarium verticillioides* (Sacc.) Nirenberg and *Fusarium proliferatum* (Matsushima) Nirenberg and are ubiquitous in U.S. maize [5]. Dietary exposure to fumonisins results in negative health impacts in humans and a variety of animal species, if the exposures exceed tolerable levels. Nearly all DDGS are consumed by beef and dairy cattle, swine, and poultry [4]. Of these species, swine are the most sensitive to dietary fumonisins. One study estimates that chronic, low-level exposure of swine to fumonisins in the form of contaminated DDGS could result in annual industry losses ranging from $9–74 million [6]. Losses are highly dependent on the fumonisin contamination of incoming grain. These estimates were based on anticipated losses due to reduced weight gain resulting from dietary fumonisin exposure in the form of contaminated DDGS included as 10%–20% total feed. This inclusion is well below the acceptable 50% inclusion of fumonisin-contaminated feed in the diet (see Table 1) but aligns with industry trends reported from 2006, where 12% of swine operations fed DDGS at an average 10% of total feed [7]. In addition to reduced weight gain in swine, outcomes of dietary fumonisin exposures exceeding tolerable levels can also result in pulmonary edema and liver damage in swine [8] and altered liver and immune function in cattle [9]. Ledoux *et al.* [10] demonstrated that the level of fumonisin contamination necessary to promote toxicity in broiler chicks is less than 100 mg/kg feed.

**Table 1:** FDA guidance levels for total fumonisins (FB_1_ + FB_2_ + FB_3_) in corn and corn by-products intended for animal consumption and maximum inclusion of contaminated feed as a percentage of the diet on a dry weight basis [11].

Species	Total Fumonisins (FB_1_ + FB_2_ + FB_3_)	Maximum Inclusion
Equids and rabbits	5 mg/kg	20%
Swine and catfish	20 mg/kg	50%
Breeding ruminants, breeding poultry, and breeding mink *	30 mg/kg	50%
Ruminants ≥ 3 months old being raised for slaughter; mink being raised for pelt production	60 mg/kg	50%
Poultry being raised for slaughter	100 mg/kg	50%
All other species or classes of livestock and pet animals	10 mg/kg	50%

* including lactating dairy cattle and hens laying eggs for human consumption.

Fumonisins are heat-stable and non-volatile under ethanol processing conditions. As a result, the “rule of thumb” for estimating fumonisin enrichment in DDGS is up to 3× the level found in the originating grain [12]. This enrichment estimate is empirically based on the typical dry-grind process which produces a mass of DDGS equal to approximately 1/3 of the mass of grain which entered the process. These DDGS are assumed to contain all of the original fumonisins—hence, threefold enrichment. Few studies have been performed to verify this enrichment factor. In one study, naturally-contaminated corn contaminated at 15 and 36 mg/kg FB_1_ was fermented and fumonisin concentrations were determined in the fractionated corn mash. The combined FB_1_ contamination of fractions which, when combined, constitute DDGS produced an enrichment ranging from 1.37× to 1.94× compared to the original maize. Interestingly, in this same study, the control maize had no detectable fumonisins in its ground form but, after fermentation, fumonisins were detected in the resultant DDGS. The study did not validate the 3× enrichment prediction, and the authors hypothesized that processing conditions may have altered the solubility of the fumonisin molecules in the extraction solvent used [12]. Two surveys of U.S. DDGS conducted from 2006–2008 and 2009–2011, reported 6 and 12% of DDGS samples, respectively, with fumonisin contamination exceeding FDA guidance levels for the most sensitive species [13,14]. Common among these two surveys and two additional studies [15,16] is a high prevalence of fumonisins in DDGS samples. Fumonisins are not the only mycotoxins subject to enrichment in DDGS; it was demonstrated for deoxynivalenol, for example, in the 2009–2011 survey previously mentioned as well as in another 2011 survey [17]. Maize may be tested for mycotoxins (including fumonisins) upon delivery at an ethanol plant if conditions have been favorable for the growth of toxigenic fungi or if mycotoxin contamination is otherwise suspected, but screening resultant DDGS is currently uncommon.

In addition to detrimental implications of fumonisin enrichment in DDGS intended for livestock feed, there is also a need to examine the impacts of grain fumonisin contamination on ethanol production itself. Strains of *Saccharomyces cerevisiae* (which are common brewing yeasts) have shown sensitivity to fumonisin B_1_, manifesting in inhibition of culture growth rates [18]. Additionally, fumonisin contamination <3 mg/kg was not found to have a significant effect on ethanol production [19] but contamination in the range of 100–1400 mg/kg resulted in a decrease in final ethanol yield with increasing fumonisin concentration [20]. At the extremes, final ethanol yield was reduced from 6% by weight (for uncontaminated maize) to 3% by weight (in 1400 mg/kg contaminated maize) in the fermentation broth [20]. Contamination at 1400 mg/kg fumonisin exceeds levels reported for naturally-contaminated maize and was achieved in this study through fungal inoculation of kernels; there currently are no results available for ethanol production from maize naturally contaminated with fumonisins in a regionally-relevant range of concentrations. The use of fumonisin-contaminated maize for fuel ethanol production poses risks worthy of further examination as they have implications for the solvency of the ethanol industry as well as health and productivity of DDGS-consuming livestock.

Transgenic insect-resistant maize (*Bt* maize) has been genetically modified to produce insecticidal proteins, which provide inherent protection from target *Lepidopteran* pests. This maize constituted more than 75% of maize grown in the U.S. in 2013 [21]. The first commercial *Bt* hybrids expressed the protein Cry1Ab, which primarily targeted the European corn borer (ECB) (*Ostrinia nubilalis* Hübner). This particular pest has been recognized as one of the most damaging maize pests in the U.S. [22]. Another *Bt* protein, Cry1F, also displays efficacy against Western bean cutworm (WBC) (*Striacosta albicosta* Smith) [23,24]. Recently, *Bt* hybrids have been developed which express proteins that effectively control additional, important maize pests including corn earworm (CEW) (*Helicoverpa zea* Boddie) [25,26]. The latter two insects have the potential to become pests of primary concern in major maize-producing regions of the U.S. [27,28]. Benefits of *Bt*-derived insect protection reach beyond the obvious yield benefits [29] and decreased physical kernel injury that results from suppression of insect feeding. When compared with conventional, non-*Bt* hybrids, hybrids expressing *Bt* genes experience reduced infection by *Fusarium* fungal species and a lower risk of contamination with fumonisin mycotoxins [30]. Although there are no data currently available to directly measure the influence, it is likely that *Bt* maize already contributes significantly to improved DDGS quality as a result of its reduced fumonisin contamination and high adoption in the United States.

Current fumonisin monitoring and control strategies in DDGS depend upon a reliable and predictable enrichment factor for mycotoxins, even though this factor lacks validation. Survey studies conducted at ethanol facilities have provided some insight into the prevalence of fumonisins in DDGS, but the significant source of uncertainty in these surveys is that a sample of DDGS cannot be traced back to its originating maize. This confounds the measurement of a direct fumonisin enrichment factor. The current study was conducted to provide more detailed information regarding fumonisin enrichment in DDGS and direct impacts of using fumonisin-contaminated maize on ethanol production efficiency. Naturally-contaminated maize was obtained from field trials in which various levels of contamination were induced by growing maize hybrids that expressed a variety of *Bt*-derived insecticidal proteins (and near-isogenic, non-*Bt* hybrids), under conditions of natural or manual insect infestation. Hybrids included in the study expressed the following insecticidal proteins: Cry1Ab (with insecticidal activity against ECB), Cry1F (active against both ECB and WBC), Cry1Ab and Vip3Aa (a combined activity against ECB, WBC, and CEW), or no insecticidal activity. Insect infestation treatments were chosen to reflect realistic species pressures in the growing area, and were also selectively suppressed by the various *Bt* or non-*Bt* hybrids used. Through the fermentation of grain obtained from these various hybrids, we seek to elucidate the important role of *Bt* maize in providing grain which is low in mycotoxins, and its influence on maximizing ethanol production and the quality of co-product DDGS.

## 2. Results

### 2.1. HPLC Method Performance

HPLC method performance and results of recovery experiments have been reported previously [24]. DDGS recovery experiments were performed for FB_1_ for five levels of spike. Results were obtained by combining the DDGS from four individual fermentations at each level of spike, and using these as one sample for HPLC determination of FB_1_. Recoveries are reported in Table 2, and ranged from 96.4%–119.2%, slightly higher than the 88% reported by Bothast *et al.* for artificially contaminated fermentations equivalent to 5 mg/kg in grain [12]. An additional set of four controls were run in the same manner (with no FB_1_ spike) and zero FB_1_ was detected in ground grain, but the DDGS contained detectable FB_1_ equivalent to 0.20 mg/kg in grain.

**Table 2:** Percent recoveries by HPLC of FB_1_ from fumonisin-spiked fermentations.

Spike Level	Concentration of FB_1_ in Grain after Spike (mg/kg)	Total FB_1_ Recovered (%)
1	7	102.2%
2	15	119.2%
3	23	96.4%
4	30	108.3%
5	37	99.9%

### 2.2. Total Fumonisins (FB_1_ + FB_2_ + FB_3_) in Ground Grain

Grain used for fermentation trials and DDGS production was obtained from field trials conducted in 2008–2011. The results of insect injury, Fusarium ear rot symptoms, and total fumonisin analysis in grain for these samples have been previously reported [24,25]. A summary of results of analyses for total fumonisins in ground grain for individual experiments conducted in each year is shown in Table 3.

**Table 3:** Mean fumonisin levels (mg/kg) in ground grain samples from experiments conducted in 2008–2011. Bold values within the experiment row denote the treatment means within that experiment. Within each experiment, means with the same superscript letter do not differ significantly (*p* ≤ 0.05) as determined by means comparison of log transformed data. Insect infestation treatments are abbreviated as follows: Nat-natural, ECB-European corn borer, WBC-Western bean cutworm, CEW-corn earworm.

Experiment and hybrid	Insect Infestation Treatment				Hybrid Means
	Nat	ECB	WBC	CEW	
**2008 Experiment A**	**1.188**	**5.240**			**3.214**
No *Bt*	0.860 ^b^	12.34 ^a^			6.598
Cry1Ab	1.446 ^b^	1.393 ^b^			1.419
Cry1F	1.257 ^b^	1.991 ^b^			1.624
**2008 Experiment B**	**1.416**	**2.120**		**3.577**	**2.371**
No *Bt*	2.317 ^ab^	4.552 ^ab^		7.756 ^a^	4.875
Cry1Ab	1.857 ^abc^	1.297 ^bc^		2.759 ^ab^	1.971
Cry1Ab/Vip3Aa	0.072 ^e^	0.512 ^cd^		0.217 ^de^	0.267
**2009 Experiment A**	**0.105**	**0.5474**	**0.553**		**0.400**
No *Bt*	0.116 ^cd^	1.245 ^a^	0.636 ^abc^		0.667
Cry1Ab	0.147 ^bcd^	0.301 ^abc^	0.780 ^ab^		0.397
Cry1F	0.041 ^d^	0.201 ^abcd^	0.226 ^abcd^		0.158
**2009 Experiment B**	**1.548**	**2.402**	**1.657**	**1.426**	**1.758**
No *Bt*	3.031 ^ab^	5.334 ^a^	2.354 ^ab^	2.418 ^ab^	3.284
Cry1Ab	1.447 ^abc^	1.331 ^bc^	2.503 ^ab^	1.609 ^abc^	1.723
Cry1Ab/Vip3Aa	0.164 ^de^	0.542 ^cd^	0.115 ^e^	0.251 ^de^	0.268
**2010 Experiment A**	**0.434**	**0.744**	**0.500**		**0.560**
No *Bt*	0.411 ^ab^	0.995 ^a^	0.842 ^a^		0.749
Cry1Ab	0.291 ^ab^	0.683 ^a^	0.535 ^a^		0.503
Cry1F	0.600 ^a^	0.555 ^a^	0.125 ^b^		0.426
**2011 Experiment B**	**0.814**	**1.159**	**1.030**	**0.819**	**0.955**
No *Bt*	1.215 ^abc^	2.714 ^a^	1.708 ^ab^	1.683 ^ab^	1.830
Cry1Ab	0.593 ^abc^	0.533 ^abc^	0.949 ^abc^	0.492 ^abc^	0.642
Cry1Ab/Vip3Aa	0.633 ^abc^	0.230 ^c^	0.432 ^abc^	0.281 ^bc^	0.394
**Insect treatment means**	**0.899**	**2.080**	**0.910**	**1.941**	**1.436**

### 2.3. Total Fumonisins (FB_1_ + FB_2_ + FB_3_) in Dried Distiller’s Grains and Solubles (DDGS)

**Figure 1:**
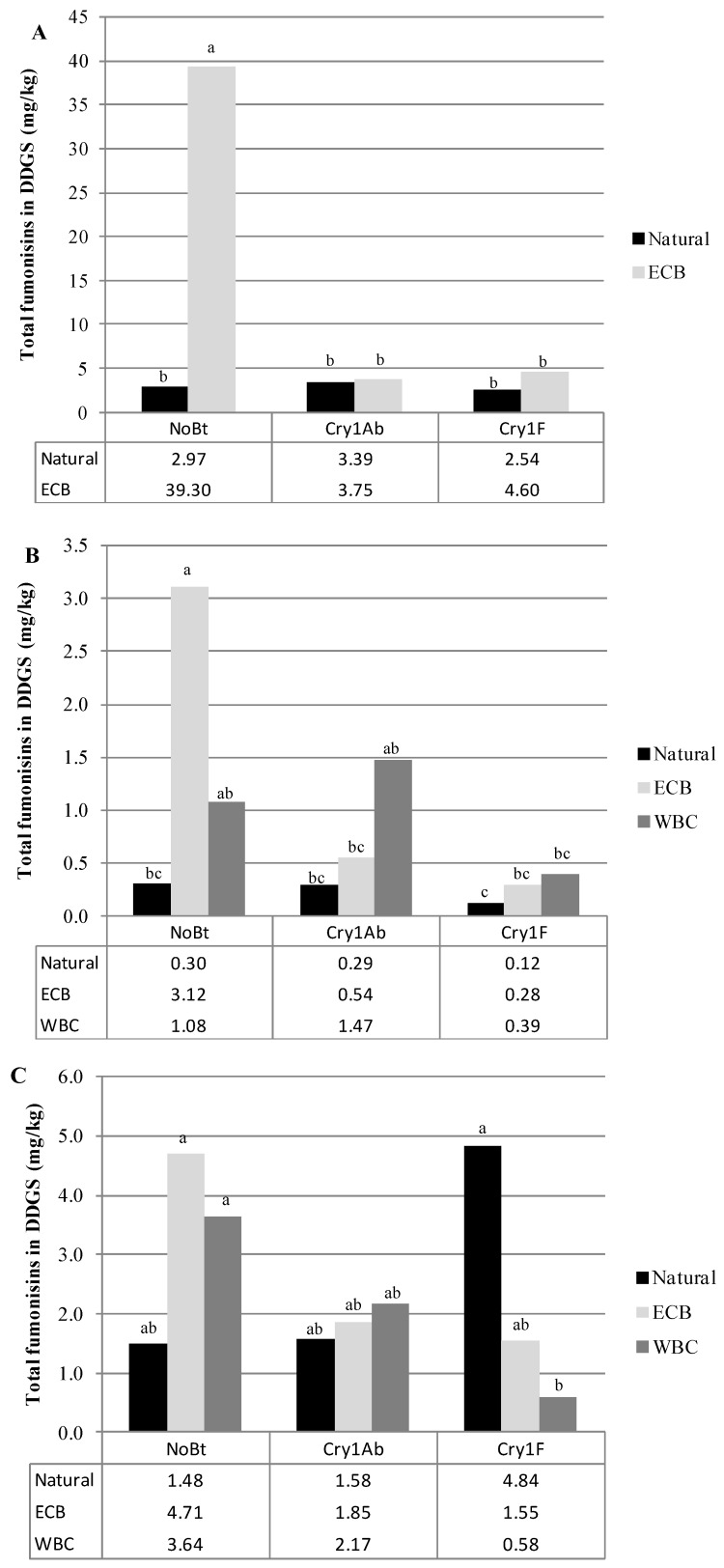
Mean total fumonisin concentration (FB_1_ + FB_2_ + FB_3_) in dried distiller’s grains and solubles (DDGS) for experiment A in (**A**) 2008; (**B**) 2009; and (**C**) 2010. Letters denote significant differences (*p* < 0.05) between hybrid/insect infestations within years.

**Figure 2:**
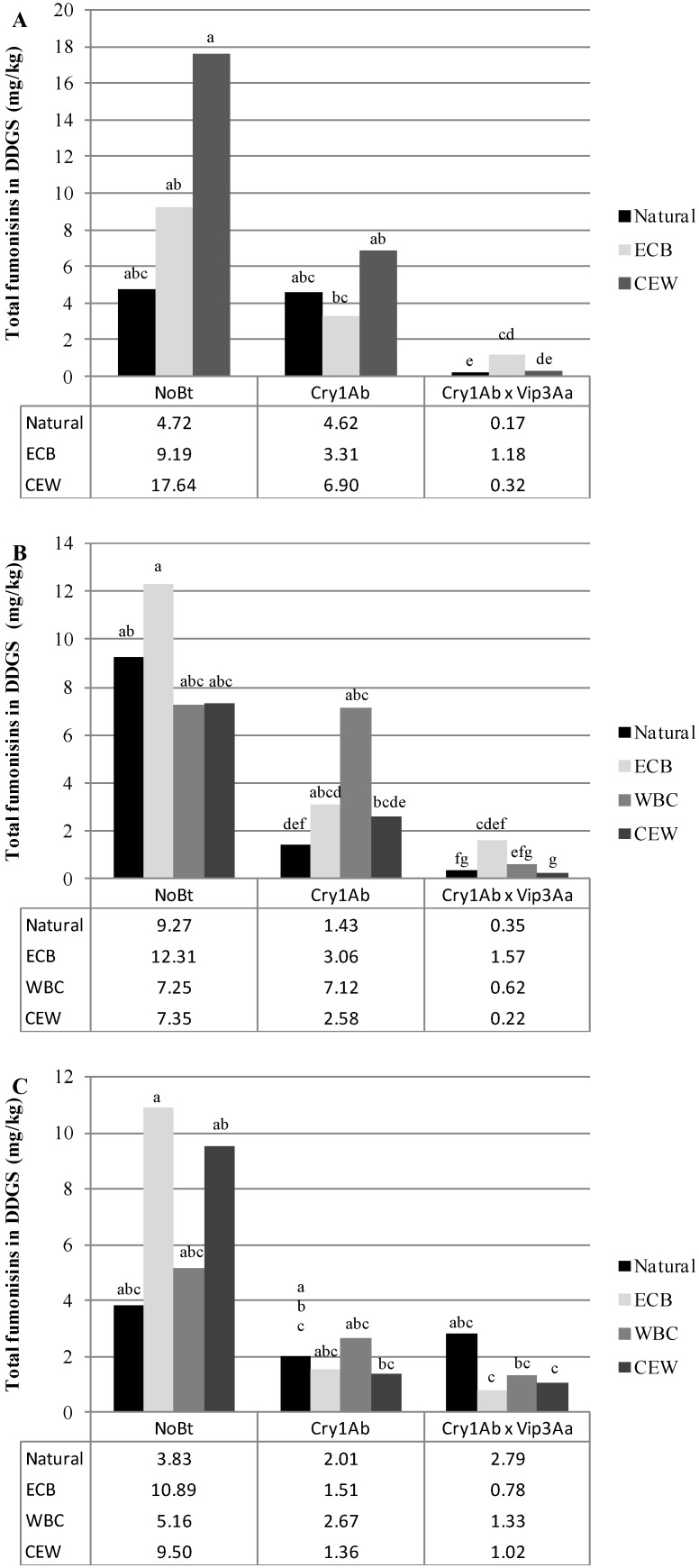
Mean total fumonisin concentration (FB_1_ + FB_2_ + FB_3_) in dried distiller’s grains and solubles (DDGS) for experiment B in (**A**) 2008; (**B**) 2009; and (**C**) 2011. Letters denote significant differences (*p* < 0.05) between hybrid/insect infestations within years.

In 2008, total fumonisin levels in DDGS were significantly influenced by hybrid, insect infestation treatment, and the interaction of these factors in both experiment A and experiment B (For experiment A, *F*_2,54_ = 6.27 and *p* = 0.0036, *F*_1,54_ = 13.18, *p* = 0.0006, and *F*_2,54_ = 6.34 and *p* = 0.0034, respectively; for experiment B, *F*_2,63_ = 83.67 and *p* < 0.0001, *F*_2,63_ = 6.16 and *p* = 0.0036, and *F*_4,63_ = 4.92 and *p* = 0.0016, respectively). In both experiments conducted in 2008, DDGS from non-*Bt* hybrids had significantly higher levels of fumonisins than DDGS from Cry1Ab hybrids (experiment A *p* = 0.0109, experiment B, *p* = 0.0319), Cry1F hybrids (*p* = 0.0080), or Cry1Ab/Vip3Aa hybrids (*p* < 0.0001). Additionally, in experiment B, fumonisin levels in DDGS derived from Cry1Ab × Vip3Aa hybrids were significantly lower than in DDGS produced from Cry1Ab hybrids (*p* < 0.0001). Naturally infested plots in experiment A had DDGS with lower fumonisin levels than ECB infested plots (*p* = 0.0006) and similarly, in experiment B, naturally infested plots were less contaminated than either ECB (*p* = 0.011) or CEW (*p* = 0.0083). Among all possible combinations of hybrid and insect treatment in any experiment, in any year, the highest levels of fumonisins in DDGS were found in those derived from ECB infested, non-*Bt* plots in 2008 experiment A (Figure 1A). Insect infestation treatments in experiment B did not differ significantly within hybrids with the exception of the Cry1Ab × Vip3Aa hybrid where DDGS from naturally infested plots had significantly lower fumonisin contamination than DDGS from ECB infested plots (*p* = 0.0012, Figure 2A). 

In 2009 DDGS fumonisin levels in experiment A were significantly affected by the hybrid (*F*_2,69_ = 9.64 and *p* = 0.0002) and insect infestation treatment (*F*_2,69_ = 10.8 and *p* < 0.0001, Figure 1B). Among the hybrids used in this experiment, those expressing Cry1F proteins had significantly less fumonisin contamination in DDGS than either Cry1Ab (*p* = 0.0116) or non-*Bt* hybrids (*p* = 0.0002). Naturally infested plots in this experiment had less contamination than either ECB (*p* = 0.0005) or WBC (*p* = 0.0004) infested plots. DDGS obtained from 2009 experiment B had fumonisin levels which were significantly affected by hybrid (*F*_2,84_ = 76.78 and *p* < 0.0001), insect infestation treatment (*F*_3,84_ = 5.73 and *p* = 0.0013), and the interaction of these two factors (*F*_6,84_ = 2.9 and *p* = 0.0129). In this particular experiment, the differences in mean DDGS fumonisin contamination among all of the hybrids were highly significant (*p* < 0.0001 for all pairwise combinations) with non-*Bt* > Cry1Ab > Cry1Ab/Vip3Aa. Among the insect infestation treatments used, DDGS produced from ECB-infested plots were more contaminated than plots with natural or CEW infestations (*p* = 0.0104 and *p* = 0.0070, respectively) but did not differ significantly from levels found in WBC-infested plots. Experiment B DDGS fumonisin levels did not differ among insect treatments within the non-*Bt* hybrid, however, naturally infested Cry1Ab plots had significantly less contamination than WBC-infested Cry1Ab (*p* = 0.0343) and CEW-infested Cry1Ab/Vip3Aa plots were significantly less contaminated than ECB-infested Cry1Ab × Vip3Aa (*p* = 0.0028, Figure 2B).

Only experiment A was conducted in 2010, and DDGS fumonisin levels were found to be influenced by the interaction of hybrid and insect infestation treatment (*F*_4,81_ = 5.09 and *p* = 0.0010). DDGS from Cry1F hybrids in the WBC infestation treatment had significantly lower fumonisin concentrations than the WBC- and ECB-infested non-*Bt* hybrids and the naturally infested Cry1F hybrids (*p* = 0.0209). In the naturally infested and ECB treatments, differences across hybrids were not significant. (Figure 1C).

In 2011 only experiment B was conducted and hybrid was a significant determinant for DDGS fumonisin contamination (*F*_2,84_ = 15.69 and *p* < 0.0001). Among the hybrids used, non-*Bt* hybrids suffered the highest levels of DDGS fumonisin contamination as compared with either Cry1Ab hybrids (*p* = 0.0002) or Cry1Ab × Vip3Aa hybrids (*p* < 0.0001). In this experiment, the highest level of DDGS contamination was found those derived from ECB-infested non-*Bt* plots (Figure 2C).

### 2.4. Fumonisin Enrichment in DDGS

The total fumonisin enrichment factors (total fumonisins in DDGS/total fumonisins in ground grain) were analyzed to determine if they differed significantly from the empirical estimate of three for each combination of hybrid × insect infestation treatment in individual experiments. In 50 of 57 individual treatment comparisons, the enrichment factor did not differ significantly from 3.0, and none of the overall experimental ratios differed from 3.0. In 2008, the enrichment factor was found to differ significantly for Cry1F × ECB (*p* = 0.0129) in experiment A, and for non-*Bt* × ECB (*p* = 0.0372) and Cry1Ab × natural (*p* = 0.0400) in experiment B. In 2009, it was found to differ significantly for Cry1Ab × WBC (*p* = 0.0135), Cry1F × ECB (*p* = 0.0074), and Cry1F × natural (*p* = 0.0372) in experiment A, and Cry1Ab × Vip3Aa × CEW (*p* = 0.0432) in experiment B (Table 4). A similar set of significant differences were identified when only FB_1_ enrichment was examined, except non-*Bt* × ECB in 2008 experiment B and Cry1F × natural in 2009 experiment A did not differ significantly from the anticipated 3× enrichment factor, and FB_1_ enrichment in non-*Bt* × natural in 2008 experiment B was significantly different (*p* = 0.0427). Twenty-six samples in the data set contained no detectable fumonisins in ground grain and of these only five remained fumonisin-free after fermentation. The remaining 21 samples averaged 0.18 mg/kg total fumonisins in DDGS.

**Table 4:** Mean enrichment factor for fumonisins from ground grain to DDGS by year, experiment, and insect treatment. Shaded means are those for which the total fumonisin enrichment factor differs significantly from the empirical factor of 3.0 (*p* ≤ 0.05). Absent insect infestation treatments in experiments are denoted with na—not available.

Experiment and hybrid	Insect Infestation Treatment				Hybrid Means
	Nat	ECB	WBC	CEW	
**2008 Experiment A**	**2.95**	**3.03**	**na**	**na**	**2.99**
No *Bt*	3.46	3.79	na	na	3.62
Cry1Ab	2.77	2.98	na	na	2.87
Cry1F	2.62	2.32	na	na	2.47
**2008 Experiment B**	**1.97**	**2.65**	**na**	**2.98**	**2.53**
No *Bt*	2.17	2.11	na	2.55	2.28
Cry1Ab	2.37	2.83	na	2.47	2.56
Cry1Ab/Vip3Aa	1.36	3.00	na	3.91	2.76
**2009 Experiment A**	**1.73**	**1.99**	**1.63**	**na**	**1.79**
No *Bt*	1.45	2.69	0.89	na	1.75
Cry1Ab	1.29	1.76	2.04	na	1.68
Cry1F	2.56	1.56	1.83	na	1.97
**2009 Experiment B**	**2.36**	**2.83**	**3.15**	**2.25**	**2.65**
No *Bt*	2.94	2.62	2.99	2.87	2.86
Cry1Ab	1.93	2.91	2.87	2.31	2.51
Cry1Ab/Vip3Aa	2.22	2.97	3.57	1.57	2.58
**2010 Experiment A**	**7.30**	**5.43**	**4.08**	**na**	**5.60**
No *Bt*	7.40	5.43	3.39	na	5.41
Cry1Ab	9.25	8.19	4.93	na	7.46
Cry1F	5.25	2.66	3.93	na	3.95
**2011 Experiment B**	**2.35**	**3.60**	**3.11**	**4.56**	**3.41**
No *Bt*	1.81	4.66	3.28	5.89	3.91
Cry1Ab	2.65	3.67	3.27	4.43	3.51
Cry1Ab/Vip3Aa	2.60	2.47	2.77	3.38	2.80
**Insect treatment means**	**3.25**	**3.30**	**3.06**	**3.26**	**3.23**

### 2.5. Impact of Fumonisin Contamination on Ethanol Yield

**Figure 3:**
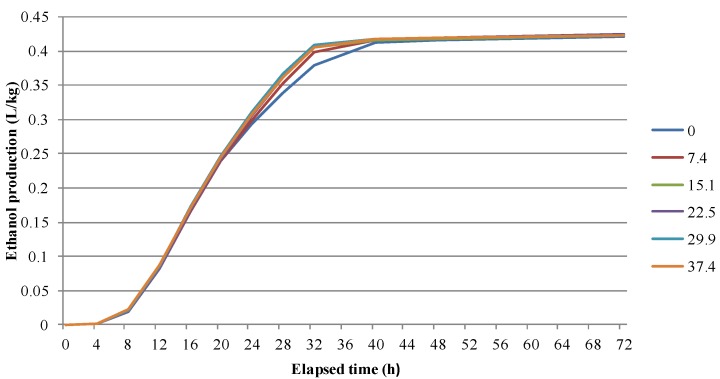
Ethanol production (L/kg) over 72 h of fermentation for grain artificially contaminated with FB_1_ in the range of 0–37 mg/kg.

In artificially-contaminated maize, there was no significant difference among the FB_1_- spike levels in the final amount of ethanol produced in the range of FB_1_ contamination tested. There were significant differences in ethanol production among spike levels between 12 and 32 h after beginning fermentation (0.0230 < *p* < 0.0001, Figure 3), however, these differences were overcome by 40 h.

In naturally-contaminated maize, there was no relationship between ethanol yield and either insect injury or Fusarium ear rot symptoms. There was also no relationship between ethanol yield and ground grain fumonisin concentrations in the range of 0–35 mg/kg with contaminated samples distributed as follows: 0 < 2 mg/kg, *n* = 384; 2 < 4 mg/kg, *n* = 69; 4 < 6 mg/kg, *n* = 14; 6 < 8 mg/kg, *n* = 7; 8 < 10 mg/kg, *n* = 9, 10 < 15 mg/kg, *n* = 4; 15 < 20 mg/kg *n* = 3; 20 < 35 mg/kg, *n* = 1. The mean ethanol yield over all experiments was 0.410 ± 0.0091 L/kg (2.75 ± 0.06 gallons/bushel) for grain at 15% moisture. In four of the six experiments, there were no significant differences in ethanol yield among hybrids. However, in experiment A in both 2008 and 2009, ethanol production differed significantly among hybrids (*F*_2,54_ = 3.66, *p* = 0.0323 and *F*_2,69_ = 7.26, *p* = 0.0014, respectively). In 2008 this difference was significant between Cry1Ab (mean 0.414 L/kg (2.78 gallons/bushel)) and Cry1F (mean 0.408 L/kg (2.74 gallons/bushel)) (*p* = 0.0244) and in 2009 there was a significant difference between Cry1F hybrids (mean 0.410 L/kg (2.75 gallons/bushel)) and both Cry1Ab (*p* = 0.0042) and non-*Bt* hybrids (*p* = 0.0041) (mean 0.414 L/kg (2.78 gallons/bushel for both).

## 3. Discussion

The results of fermentation of both naturally and artificially fumonisin-contaminated maize suggest that ethanol yields per kg of grain are not likely to be affected by naturally occurring fumonisin contamination in the Central United States. The ranges of kernel injury, Fusarium ear rot, and grain fumonisin concentrations in this study are representative of maize production in the region. Treatment means ranged as high as 12.3 mg/kg in total fumonisins and individual samples ranged as high as 34.9 mg/kg. Typical fumonisin concentrations in maize grain rarely exceed these levels under natural conditions in most maize-growing areas. Within this range, there were no consistent relationships between ethanol yield and insect injury, infection, or ground grain fumonisin contamination. Sosa *et al.* did find fumonisin contamination impacted ethanol yield, however the levels of contamination at which this occurred were unrealistic for naturally contaminated maize in the U.S. (100–1400 mg/kg) [20]. ANOVA analysis performed on mean ethanol production for the hybrid × insect infestation combinations in the six experiments revealed only a few significant differences in ethanol yield among the different treatment combinations, and these differences were always <1.5% of total ethanol yield. In 2008 and 2009 there were significant differences in ethanol production among hybrids but they did not correspond with differences in fumonisin contamination among hybrids. Within the range of natural fumonisin contamination found in grain used in this study, there was no detectable effect of grain fumonisin contamination on final ethanol production; nor did the occurrence of insect injury or Fusarium ear rot have a significant impact on ethanol yield (within the ranges of severity that occurred). Although ethanol production was unaltered with respect to grain quality in the current study, in either naturally or artificially contaminated maize, the 72 h fermentation time is longer than that used in most commercial ethanol processes. In commercial settings, fermentation time generally does not exceed 64 h and can be as little as 50 h in attempts to maximize efficiency. As previously discussed, one of the prominent concerns with mycotoxin contamination in fermentation is their detrimental effect on viability and reproduction of *S. cerevisiae* [18,31]. The current study utilized a high initial loading of yeast (0.5 g/25 g maize) in fermentations of naturally contaminated grain; however, there was no significant effect on ethanol yield even when yeast concentration was reduced to 0.025 g/25 g maize in the artificially spiked fermentation. This suggests that toxicity of fumonisins to *S. cerevisiae* did not impact yeast activity under the fermentation conditions utilized, for maize contaminated in the range of 0–37 mg/kg fumonisins. Any hindrance in yeast activity or reproduction which may result from fumonisin contamination were not detectable in the final ethanol yield after 72 h of fermentation (used in this study); in commercial settings with shorter fermentation times, there could be ethanol yield impacts if rates of yeast reproduction and/or activity are initially suppressed and the effect is not sufficiently overcome in these shorter fermentation times. The results from the artificially fumonisin-contaminated fermentation indicated that effects on fermentation rate, if any, were overcome as early as 40 h after the start of the fermentation.

Although in this study, ethanol yield per kg of grain was not consistently different between *Bt* and non-*Bt* hybrids, the yield of ethanol per unit land area is estimated to be greater on average for *Bt* hybrids. Yield benefits of growing *Bt* maize hybrids in the U.S. (as compared with conventional, non-*Bt* maize) have been estimated at 1067 kg/hectare (15% moisture basis, 17 bushels/acre) [29]. Considering the average ethanol yield measured in this study, 0.410 ± 0.0091 L/kg (2.75 ± 0.06 gallons/bushel), a maize yield difference of 1067 kg/hectare is equivalent to a difference in ethanol production potential of 438 L/hectare (47 gallons/acre) between *Bt* and non-*Bt* maize acres. In addition to potential gains in ethanol productivity per unit land area, this study has shown that *Bt* maize hybrids produce higher quality DDGS as compared with non-*Bt* DDGS, from the standpoint of fumonisin mycotoxin contamination. The average fumonisin content of *Bt*-derived DDGS in this study was 2.04 mg/kg as compared with DDGS derived from non-*Bt* hybrids which averaged 8.25 mg/kg. Within the *Bt* hybrids tested, those with broader spectra of insecticidal activity had lower mean fumonisin contamination levels. Cry1Ab targets only ECB and DDGS derived from these maize hybrids had an average fumonisin concentration of 2.64 mg/kg. Cry1F targets ECB and WBC and these DDGS averaged 1.97 mg/kg while Cry1Ab/Vip3Aa targets ECB, WBC, and CEW and these DDGS averaged 0.94 mg/kg. *Bt* maize fermentations produced DDGS, which were consistently lower in fumonisin contamination than DDGS derived from non-*Bt* maize. These reduced levels of contamination increase the range of livestock which can consume them without experiencing negative health effects and provide greater flexibility for DDGS inclusion rates in livestock diets. The utilization of *Bt* maize for fermentation maximizes the marketability of DDGS co-product, and ensures optimization of animal health and productivity for animals that consume them as a portion of their diet.

Our results are consistent with the typical assumption of a threefold enrichment for mycotoxin content in DDGS compared to the original grain. Bennett *et al.* [32] examined the impacts of using zearalenone-contaminated grain for ethanol production and found that, like fumonisins, no toxins were recovered in ethanol fractions; rather, they were recovered in fermentation solids at approximately twice the concentration in the original grain. Schaafsma *et al.* sampled grain and DDGS at a commercial ethanol facility and found deoxynivalenol concentrations approximately three times higher in DDGS than in the incoming grain [33]. The current study has provided laboratory-scale results which support this threefold enrichment factor for fumonisins in DDGS. The highest enrichment factors were found in 2010 (experiment A), but these did not differ significantly from the expected 3× factor due to their large standard deviations. The average fumonisin contamination in ground grain for these samples was relatively low (≤1 mg/kg) and DDGS produced from this grain had highly variable fumonisin contamination, but the means still fell below the lowest FDA guidance level for fumonisin-contaminated feed. According to the statistical comparisons, 50 out of the 57 hybrid × insect treatment combinations had enrichment factors that were not significantly different from 3.0. The seven instances where the enrichment factors differed from 3.0 all occurred in 2008 and 2009. For all seven of these instances, the mean enrichment factor was <3.0, averaging as little as 1.56 in ECB infested Cry1F hybrids in 2009 experiment A. The average fumonisin contamination in DDGS for each of these seven exceptions was below 10 mg/kg and five of these exceptions did not exceed 5 mg/kg. The examples of enrichment factors significantly lower than 3.0 may have been due to masking of fumonisins in the DDGS matrix.

Masked fumonisins (and masked mycotoxins in general) have come to the attention of scientists and are a significant concern for food and feed safety. Masked mycotoxins are undetectable by many of the common analytical methods due to structural alterations occurring *in planta* [34,35,36] or during processing. Some examples of modifications which mask the toxins are removal or alteration of functional groups and covalent or non-covalent binding of the mycotoxins to matrix components. Masked fumonisins have been found to occur in heat-treated food products [37]. Dall’Asta *et al.* have reported the occurrence of hidden fumonisins in raw maize, hypothesizing that this is a result of non-covalent associations with matrix macromolecules [38]. Whether altered in the plant or during processing, masked mycotoxins may enter food and feed chains undetected in contaminated grains. Through conditions incurred in grain handling and processing or through metabolic transformations occurring after consumption, they can be made bioavailable in their original form or rendered more toxic through physical and/or chemical transformations. Currently, there is little if any data on the toxicological effects of masked fumonisins and identifying them in dynamic systems like maize fermentations is complex. In one study, a strain of *Saccharomyces cerevisiae* at cell densities of 2.4 × 10^7^ cells/mL was capable of binding approximately 60% of the FB_1_ in solution, even at concentrations of 100 ug/mL FB_1_. This is especially interesting in relation to maize ethanol production as this *S. cerevisiae* cell density is similar to the density used in maize ethanol fermentation (2.0 × 10^7^ cells/mL). *S. cerevesiae* cells were capable of FB_1_ binding regardless of cell viability [39] and the authors proposed the binding mechanism to be physical adsorption of fumonisins to cell wall components. Additionally, *Lactobacillus* bacteria are common contaminants of maize ethanol facilities [40] and some of these bacterial strains have been shown to have the capability to generate masked fumonisins [39,41]. It is plausible that due to the processing steps incurred in generating DDGS-including heat treatment, yeast inclusion, and propensity for bacterial contamination-that masking of fumonisins may be occurring. To our knowledge, an analysis of the occurrence and identity of masked fumonisins in fermentation co-products has not been conducted; this may provide insight into the high variability among individual fermentations as well as the low fumonisin enrichment factors in some of the DDGS samples produced in this study.

In years when conditions are favorable for fumonisin-producing Fusarium species to grow and produce fumonisins, monitoring incoming grain at ethanol facilities may occur with regularity. This is typically considered to be the point of control for DDGS, as monitoring DDGS fumonisin content post-fermentation is not routine practice in ethanol facilities. The current study identified samples of ground grain without detectable fumonisins that produced DDGS containing fumonisins, albeit at low levels. Similar observations are recorded in a study conducted by Bothast *et al.* [12]. In the current study, all of these instances had a final total fumonisin concentration in DDGS that did not exceed 1 mg/kg. It is likely that fumonisins were present in these ground grain samples, but at levels below the limit of detection, and were enriched in DDGS post-fermentation to a level above the limit of detection. The current study also provides laboratory-scale validation for the assumption of a threefold enrichment of fumonisin from ground grain to DDGS, and poses some new questions regarding fumonisin stability and potential masking during fermentation. Seven of 57 calculated enrichment factors were significantly lower than the anticipated factor of 3.0. Evidence that *S. cerevesiae* can adsorb FB_1_ [38] provides one example of a matrix effect which may render FB_1_ undetectable by conventional methods. The recognition of masked mycotoxins in grains suggests there may be mycotoxin risks even when grain is deemed free of mycotoxins by conventionally accepted analyses. Mycotoxins may still be present, bound either reversibly or irreversibly to matrix components, or structurally altered in a way that they escape conventional detection methods. These forms are poorly characterized and have the potential to become bioavailable after consumption. A greater understanding of the occurrence and identity of masked fumonisins in fermentation processes, as well as in grains and grain-based products in general, is a growing need for food and feed safety.

## 4. Experimental Section

### 4.1. Sample Acquisition

Grain was obtained from two sets of field experiments (“A” and “B”) that were conducted in 2008–2011 in Central Iowa (Story and Boone Counties) to assess *Lepidopteran* insect infestation in maize hybrids expressing varying *Bt* transformation events. The experiments were organized as randomized complete block designs; experiment “A” consistently had 5 replicate blocks of each combination of hybrid and insect treatment while experiment “B” had 8, 8, and 4 replicates in 2008, 2009, and 2011, respectively. A summary of the field trials is provided in Table 5. In all experiments, ten primary ears from the insect-treated rows of each plot were harvested by hand. Ears were stored in a cold room (4 °C) until drying to inhibit fungal metabolism. Post -harvest handling included visual assessment of ears for insect injury and Fusarium ear rot severity and drying at approximately 100–120 °F to 15% moisture content (dry basis). In all experiments, ears were shelled using a hand-crank sheller and kernels from the ten harvested ears per plot were combined and ground on the finest particle size setting using a Romer mill (Romer Laboratories Inc., Union, MO, USA).

**Table 5:** Summary of 2008–2011 field trials. Insect infestation treatment abbreviations are as follows: ECB—European corn borer (*Ostrinia nubilalis*); WBC—Western bean cutworm (*Striacosta albicosta*); CEW—corn earworm (*Helicoverpa zea*); Natural—no insects applied.

Year	Experiment	Location	Hybrids	Insect Infestation Treatments	Planting Date	Harvest Date
2008	A	Story County	6 total	ECB Natural	14 May	28 October
			2 Cry1F			
			2 Cry1Ab			
			2 non-*Bt*			
2008	B	Boone County	3 total	ECB Natural CEW	18 June	13 November
			1 Cry1Ab			
			1 Cry1Ab/Vip3Aa			
			1 non-*Bt*			
2009	A	Story County	6 total	ECB Natural WBC	8 May	30 October
			2 Cry1F			
			2 Cry1Ab			
			2 non-*Bt*			
2009	B	Boone County	3 total	ECB Natural WBC CEW	4 June	10 November
			1 Cry1Ab			
			1 Cry1Ab/Vip3Aa			
			1 non-*Bt*			
2010	A	Story County	6 total	ECB Natural WBC	21 April	5 October
			2 Cry1F			
			2 Cry1Ab			
			2 non-*Bt*			
2011	B	Story County	6 total	ECB Natural WBC CEW	16 May	24 October
			2 Cry1Ab			
			2 Cry1Ab/Vip3Aa			
			2 non-*Bt*			

### 4.2. Fermentation

#### 4.2.1. Reagents

Calcium chloride (200 mM) solution was prepared by dissolving 2.94 g CaCl_2_·2H_2_O (Fisher Scientific, Fair Lawn, NJ, USA) in 97.06 g distilled water and filter sterilized. Yeast nutrient was made by dissolving 6 g (NH_4_)_2_SO_4_ (Fisher Scientific, Fair Lawn, NJ, USA) in 50 mL distilled water [42]. Alpha-amylase (Spezyme RSL) and glucoamylase (Distillase SSF) were provided by Genencor (DuPont Danisco, Palo Alto, CA, USA). The yeast used was Red Star active dry yeast (Lesaffre Yeast Corporation, Milwaukee, WI, USA). With the exception of yeast nutrient (stored at room temperature), all reagents were stored at 4 °C when not in use.

#### 4.2.2. Procedure

Ethanol production was determined gravimetrically for grain obtained from each harvested plot in every year of the experiment. Briefly, 25 g ± 0.050 g of ground grain was mixed with ~72 mL distilled water in a 125-mL Erlenmeyer flask. To the flask were added, 1 mL of 200 mM CaCl_2_ solution, 400 μL yeast nutrient, 100 μL α-amylase, and a stir bar. The flasks were capped with aluminum foil and heated on a double boiler with stirring for 15 min. Flasks were then removed to a 70 °C Isotemp 120 water bath (Fisher Scientific, Fair Lawn, NJ, USA) (scraping the inside of the flask down so that all particulates were in solution), followed by a 50 °C water bath, and, finally, to room temperature, allowing enough time in each to cool the flask contents to the temperature of the specified water bath. Once the flask contents were below 40 °C, 0.5 g yeast and 0.5 mL glucoamylase were added to each flask. After these additions were made, flasks were immediately capped with a rubber stopper vented with an 18-gauge needle (to allow for gas dissipation), weighed, and a start time and weight were recorded. Flasks were then placed on an Innova 4300 incubated shaker (New Brunswick Scientific Co., Inc., Enfield, CT, USA) at 32 °C and 175 rpm for 72 h to ensure complete fermentation. All flasks being run on a given day typically had start times within 20 min of each other. Flasks were stirred once between 24 and 48 h to agitate fine particles which had settled to the bottom of the flask. End weights of flasks were recorded after the 72 h fermentation (±10 min from the recorded start time) was complete.

#### 4.2.3. Ethanol Determination

The production of carbon dioxide on a molecular scale is equivalent to the production of molecular ethanol according to Equation (1). Ethanol was determined gravimetrically by calculating carbon dioxide production (the difference in the starting and ending mass of a fermentation flask) and converting this, using molecular masses and the weight of maize fermented, to the mass of ethanol produced per unit fermented maize. Ethanol yields reported in this paper are adjusted to the standard 15% grain moisture.

Equation (1): Glucose fermentation products

Glucose (C_6_H_12_O_6_) → 2 Ethanol (C_2_H_5_OH) + 2 Carbon Dioxide (CO_2_) (1)

#### 4.2.4. DDGS Collection

Ethanol and excess water were evaporated out of the fermentation flasks by heating the flasks at 100 °C in a water bath. Final drying of the fermented mash to obtain DDGS was accomplished using a stainless steel food dehydrator at approximately 100 °C (The Sausage Maker, Inc., Buffalo, NY, USA), and dried DDGS were stored at −23 °C until HPLC analysis for fumonisin content.

#### 4.2.5. Fumonisin B_1_ Impact on Ethanol Production

Crystalline FB_1_ (BioPure, Tulln, Austria) was reconstituted in deionized water to attain a solution concentration of 1 mg/mL. Pre-weighed flasks of uncontaminated grain (provided by DuPont Pioneer, Johnston, Iowa, USA) were spiked with this FB_1_ solution to achieve artificial contamination levels equivalent to 0, 7, 15, 23, 30, and 37 mg/kg FB_1_. Fermentations were performed as described above, with the exception of yeast quantity. Yeast addition at 0.025 g/25 g maize was previously determined to be the lowest relative addition level which produced no significant effect on final ethanol yield as compared with fermentations using a standard addition of 0.5 g/25 g maize. This lower level was used for this experiment so as to capture any potential impacts on fermentation which would arise from FB_1_ toxicity to yeast. Four replicate samples were fermented at each level of contamination. DDGS resulting from the quadruplicate runs were aggregated for extraction and HPLC fumonisin determination.

### 4.3. HPLC Analysis

The HPLC method and validation have previously been reported [24]. The only adjustment from this method is in the sample preparation step, 5 g of DDGS were extracted rather than 10 g of ground grain. Recovery experiments were performed on aggregate DDGS samples obtained from artificially-contaminated maize at five levels ranging from 7 to 37 mg/kg FB_1_ in ground grain, plus a zero-contamination control (method described in Section 4.2.5).

#### HPLC Instrumentation and Parameters

The LC system consisted of a Varian ProStar 210 pump, 410 AutoSampler, and 363 fluorescence detector (Agilent Technologies, Santa Clara, CA, USA). The LC column was a Zorbax Eclipse Plus-C18, 3.5 μm (4.6 × 100 mm) and was preceded by a Zorbax Eclipse Plus-C18, 5 μm (4.6 × 12.5 mm) guard column (Agilent Technologies, Santa Clara, CA, USA). Fumonisins were eluted isocratically in a mobile phase consisting of 52:47:1 (*v*/*v*/*v*) filtered deionized water/acetonitrile/glacial acetic acid at a flow rate of 2.0 mL/min. Fumonisin-NDA derivatives were detected using 420 nm excitation and 500 nm emission wavelengths. The autosampler was run with constant tray cooling at 4 °C and column oven 30 °C.

### 4.4. Statistical Analysis

Analysis of variance (ANOVA) was performed on log-transformed fumonisin and ethanol data using the PROC GLIMMIX procedure in SAS Version 9.3 software (SAS Institute Inc., Cary, NC, USA) fitting the data to a gamma distribution. Factorial analysis was used to determine simple effects of maize event, insect infestation treatment, and their interaction. Where interactions were present, multiple comparison analysis was performed using a Tukey-Kramer adjustment. Data were analyzed as six individual experiments, as combining the data from the experiments (hybrids and insect treatments differed in experiment A and B) resulted in absent marginal means. The log of the enrichment factor for each hybrid/insect treatment combination within individual experiments was tested against a null hypothesis equal to log(3) using the UNIVARIATE procedure to identify those that differed from the empirical 3× assumption. The independent variable “hybrid” used for analysis combines results from pairs of hybrids used in the experiments which produced the same *Bt* insecticidal protein (either Cry1F, Cry1Ab, Cry1Ab/Vip3Aa, or none).

## 5. Conclusions

This study provides the first laboratory-scale validation of the threefold assumption used in industry to predict fumonisin enrichment in DDGS. In 50/57 instances, total fumonisins (FB_1_ + FB_2_ + FB_3_) in DDGS were three times the level found in the maize they originated from. In the seven instances that did not agree with this enrichment assumption, the enrichment factor was less than three. Further research is needed to establish why these few samples differed from the threefold assumption. Final ethanol yield was unaffected by regionally-relevant fumonisin concentrations used in this study (0–35 mg/kg) as was the rate of ethanol production in artificially contaminated maize (0–37 mg/kg). There also was not a consistent relationship between ethanol yield and insect injury or Fusarium ear rot severity. It is unlikely that fumonisin contamination alone would significantly impact industrial-scale ethanol yields, at least not with the levels of contamination currently occurring in the major maize-producing regions of the United States. This is not grounds to assume mycotoxins have no impact ethanol yields or production efficiency, especially if fermentation times are shortened to maximize productivity. Ethanol yields did not differ significantly between *Bt* and non-*Bt* maize hybrids, however, current grain yield estimates indicate ethanol yield per acre of maize is increased in *Bt* maize as a result of higher grain yields than non-*Bt* maize. *Bt* maize also produced DDGS which were consistently lower in fumonisin content as compared to non-*Bt* maize. Lower fumonisin levels increase the versatility of DDGS for feeding to a wider variety of livestock.
